# Self-Reported Compliance With Personal Preventive Measures Among Chinese Factory Workers at the Beginning of Work Resumption Following the COVID-19 Outbreak: Cross-Sectional Survey Study

**DOI:** 10.2196/22457

**Published:** 2020-09-29

**Authors:** Yihang Pan, Yuan Fang, Meiqi Xin, Willa Dong, Liemin Zhou, Qinghua Hou, Fanping Li, Gang Sun, Zilong Zheng, Jinqiu Yuan, Zixin Wang, Yulong He

**Affiliations:** 1 Big Data Center The Seventh Affiliated Hospital Sun Yat-sen University Shenzhen China; 2 Precision Medicine Center, Scientific Research Center The Seventh Affiliated Hospital Sun Yat-sen University Shenzhen China; 3 Department of Early Childhood Education The Education University of Hong Kong Hong Kong China (Hong Kong); 4 JC School of Public Health and Primary Care Chinese University of Hong Kong Hong Kong China (Hong Kong); 5 Department of Health Behavior Gillings School of Global Public Health University of North Carolina at Chapel Hill Chapel Hill, NC United States; 6 Department of Neurology The Seventh Affiliated Hospital Sun Yat-sen University Shenzhen China; 7 Department of Endocrinology The Seventh Affiliated Hospital Sun Yat-sen University Shenzhen China; 8 Department of Clinical Nutrition The Seventh Affiliated Hospital Sun Yat-sen University Shenzhen China; 9 Clinical Research Center The Seventh Affiliated Hospital Sun Yat-sen University Shenzhen China; 10 Center for Digestive Disease The Seventh Affiliated Hospital Sun Yat-sen University Shenzhen China

**Keywords:** COVID-19, work resumption, factory workers, facemask wearing, hand hygiene, physical distancing, prevention, cross-sectional, online, survey, compliance

## Abstract

**Background:**

Maintaining compliance with personal preventive measures is important to achieve a balance of COVID-19 pandemic control and work resumption.

**Objective:**

The aim of this study was to investigate self-reported compliance with four personal measures to prevent COVID-19 among a sample of factory workers in Shenzhen, China, at the beginning of work resumption in China following the COVID-19 outbreak. These preventive measures included consistent wearing of face masks in public spaces (the workplace and other public settings); sanitizing hands using soap, liquid soap, or alcohol-based hand sanitizer after returning from public spaces or touching public installations and equipment; avoiding social and meal gatherings; and avoiding crowded places.

**Methods:**

The participants were adult factory workers who had resumed work in Shenzhen, China. A stratified two-stage cluster sampling design was used. We randomly selected 14 factories that had resumed work. All full-time employees aged ≥18 years who had resumed work in these factories were invited to complete a web-based survey. Out of 4158 workers who had resumed work in these factories, 3035 (73.0%) completed the web-based survey from March 1 to 14, 2020. Multilevel logistic regression models were fitted.

**Results:**

Among the 3035 participants, 2938 (96.8%) and 2996 (98.7%) reported always wearing a face mask in the workplace and in other public settings, respectively, in the past month. However, frequencies of self-reported sanitizing hands (2152/3035, 70.9%), avoiding social and meal gatherings (2225/3035, 73.3%), and avoiding crowded places (1997/3035, 65.8%) were relatively low. At the individual level, knowledge about COVID-19 (adjusted odds ratios [AORs] from 1.16, CI 1.10-1.24, to 1.29, CI 1.21-1.37), perceived risk (AORs from 0.58, CI 0.50-0.68, to 0.85, CI 0.72-0.99) and severity (AOR 1.05, CI 1.01-1.09, and AOR 1.07, CI 1.03-1.11) of COVID-19, perceived effectiveness of preventive measures by the individual (AORs from 1.05, CI 1.00-1.10, to 1.09, CI 1.04-1.13), organization (AOR 1.30, CI 1.20-1.41), and government (AORs from 1.14, CI 1.04-1.25, to 1.21, CI 1.02-1.42), perceived preparedness for a potential outbreak after work resumption (AORs from 1.10, CI 1.00-1.21, to 1.50, CI 1.36-1.64), and depressive symptoms (AORs from 0.93, CI 0.91-0.94, to 0.96, CI 0.92-0.99) were associated with self-reported compliance with at least one personal preventive measure. At the interpersonal level, exposure to COVID-19–specific information through official media channels (AOR 1.08, CI 1.04-1.11) and face-to-face communication (AOR 0.90, CI 0.83-0.98) were associated with self-reported sanitizing of hands. The number of preventive measures implemented in the workplace was positively associated with self-reported compliance with all four preventive measures (AORs from 1.30, CI 1.08-1.57, to 1.63, CI 1.45-1.84).

**Conclusions:**

Measures are needed to strengthen hand hygiene and physical distancing among factory workers to reduce transmission following work resumption. Future programs in workplaces should address these factors at multiple levels.

## Introduction

**Figure 1:**
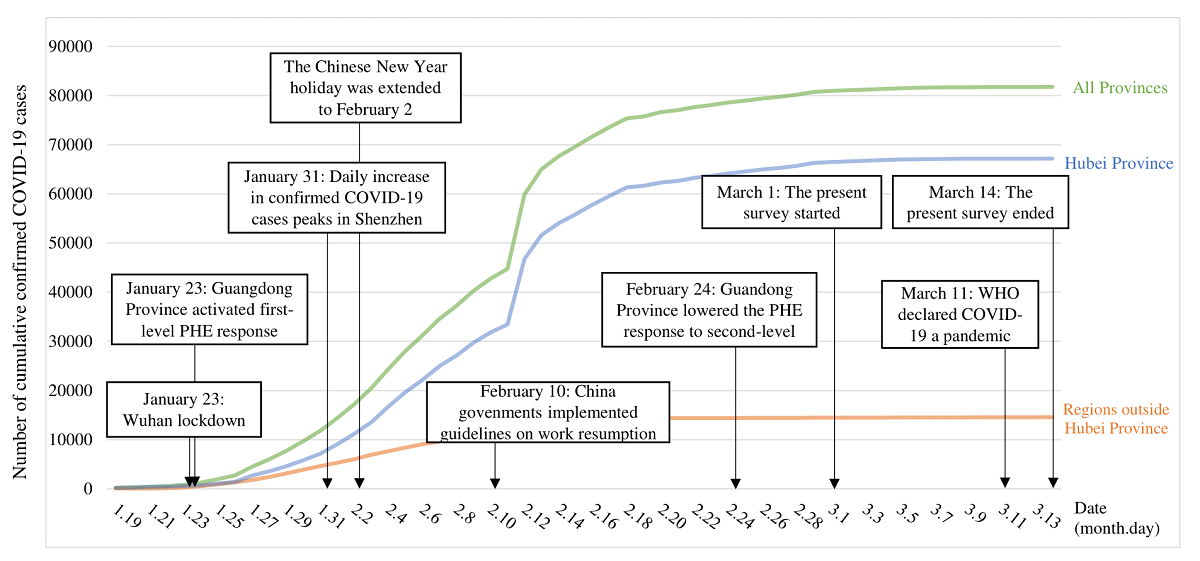
Background of the present survey, including the trend of cumulative confirmed COVID-19 cases in mainland China and critical responses to COVID-19 in Shenzhen, a city in Guangdong Province. PHE: public health emergency; WHO: World Health Organization.

As of July 1, 2020, 10,357,662 cases of COVID-19 and 508,055 deaths from the disease have been reported worldwide [1]. China has reported 85,232 confirmed COVID-19 cases and 4648 deaths [1]. To curb the epidemic, the Chinese government formally requested that enterprises not resume work prior to February 10, 2020, with the exception of those involved in providing basic and essential services [2,3]. These strict control measures were shown to be effective but were likely detrimental to the economy [1], as China reported a 6.8% decline in its first quarter gross domestic product in 2020 compared to the previous year [4] (Figure 1).

In China, full work resumption is imminent. Starting on February 10, 2020, the Chinese government implemented guidelines to ensure that enterprises were adequately prepared for work resumption. Each enterprise was required to establish a comprehensive contingency plan, appoint a designated coordinator, monitor the health status of all employees and their travel history, and ensure the supply of all necessary preventive equipment [2,3]. Local governments are assessing these preparations and granting official permission for work resumption [2,3]. To scale up work resumption, official permission from local governments was no longer required as of February 20, 2020, in some Chinese cities (eg, Shenzhen) [2]. There are concerns that an increase in public contact after work resumption may result in a second wave of the COVID-19 pandemic in China [5].

Maintaining compliance with personal preventive measures plays an important role in achieving the balance between pandemic control and work resumption. Universal use of face masks [6], hand hygiene [7], and physical distancing (eg, avoiding social and meal gatherings and avoiding crowded places) [8] are strongly advocated by the World Health Organization (WHO) and have been implemented worldwide [9,10]. The effectiveness of these personal preventive measures is crucially dependent on compliance by the public [11]. Studies conducted in China, Australia, and Thailand consistently supported that achieving very high compliance (80%-95%) with personal preventive measures has been important to control the COVID-19 pandemic in these countries [5,9,12].

Understanding factors associated with compliance with personal preventive measures is important to develop effective interventions. As interventions addressing factors at multiple levels are more likely to be successful in changing behavior, we used the socio-ecological model as the conceptual framework of our study [13]. This model considers determinants of health behaviors at the individual, interpersonal, and social-structural levels. Prior research on COVID-19 and other pandemics suggests the applicability of the socio-ecological model to inform behavioral change interventions in China. At the individual level, being knowledgeable about COVID-19 was associated with higher adoption of personal preventive measures among Hong Kong Chinese residents in the early phase of the pandemic [14]. Perceptions related to COVID-19 may also affect compliance with these personal preventive measures. For example, risk perception, perceived severity of the disease, perceived effectiveness of the preventive measures, and perceived preparedness of health systems and governments were associated with adoption of personal preventive measures during the severe acute respiratory syndrome (SARS) and H1N1 pandemics in China [15-18]. In addition, mental health status may be a particularly salient individual-level factor, as early studies in China have documented high levels of psychological problems (eg, stress, panic, depression, and anxiety) triggered by the COVID-19 pandemic [19-21]. Mental health problems have been associated with lower adoption of personal preventive measures during the COVID-19 pandemic [19]. At the interpersonal level, the heightened level of governmental alerts was accompanied by widespread coverage of COVID-19–related information across different media, including television, newspapers, and social media [14]. Additionally, different media channels may have varying effects on compliance with personal preventive measures. During the Middle East respiratory syndrome (MERS) outbreak, increased exposure to MERS-specific information through social media and interpersonal communication was associated with higher adoption of personal preventive measures. However, the association between exposure to information disseminated through traditional media (eg, television and newspapers) and personal preventive measures was nonsignificant [22,23]. At the social-structural level, implementation of organizational preventive measures during work resumption may differ across factories, which may also affect compliance with personal preventive measures.

To the best of our knowledge, no study has investigated self-reported compliance with personal preventive measures and associated factors among workers who resumed work during the COVID-19 pandemic. To address these gaps, this study investigated self-reported compliance with four personal preventive measures among a sample of factory workers in Shenzhen, China. We examined the effects of sociodemographic factors, individual-level factors (knowledge, perception, and depressive symptoms), interpersonal-level factors (exposure to COVID-19–specific information through different media), and social-structural–level factors (preventive measures implemented by the factories).

## Methods

### Study Design

We conducted a closed cross-sectional web-based survey of 3035 factory workers in Shenzhen, China from March 1 to 14, 2020. Of the 13 million residents in Shenzhen in 2018, 65.1% were internal migrants and 34.3% were factory workers [24].

### Participants and Data Collection

By March 1, 2020, 100 factories in Shenzhen had resumed work. A stratified two-stage cluster sampling design was used to recruit the study participants. First, 14 factories were randomly selected by the research team. Of these 14 factories, 10 (71%) manufactured electronic devices, 2 (14%) manufactured watches, 1 (7%) manufactured beverages, and 1 (7%) manufactured biotechnology products. All full-time employees aged ≥18 years who had resumed work in these factories were invited to complete a web-based survey.

We developed a web-based questionnaire using Questionnaire Star, a commonly used web-based survey platform in China, and the link to the questionnaire could be shared using the WeChat social media platform. In addition to national guidelines, the Shenzhen government requested that each factory establish WeChat groups including all employees as part of the preparation for work resumption [2,3]. A designated coordinator responsible for COVID-19 control in each factory facilitated the data collection. This coordinator posted the study information and the link to access the web-based self-administered questionnaire in the WeChat group, and they invited all eligible workers who had resumed work to participate. The coordinator also sent out reminders in the WeChat groups biweekly during the recruitment period. These designated coordinators did not participate in the actual survey. The coordinators and participants were asked not to disseminate the link to access the survey to people outside the 14 selected factories. Before starting the web-based survey, the participants read a statement indicating that participation was voluntary, refusal to participate would have no effect on them, the survey would not collect personal contacts or identifying information, and the data would be kept strictly confidential and would only be used for research purposes. Web-based informed consent was obtained. Each individual WeChat account was allowed to access the web-based questionnaire once to avoid duplicate responses. The survey contained 93 items (approximately 15 items per page for 6 pages) and required approximately 20 minutes to complete. The Questionnaire Star tool performed completeness checks before the questionnaire was submitted. Participants were able to review and change their responses using a Back button. An electronic coupon for ¥10 (US $1.3) was sent to participants upon completion. All data were stored in the Questionnaire Star server and protected by a password. Only the corresponding authors had access to the database. Ethics approval was obtained from the Seventh Affiliated Hospital, Sun Yat-sen University (reference: KY-2020-005-001).

### Measures

#### Design of the Questionnaire

A panel consisting of two public health researchers, a health psychologist, two clinicians, a senior factory manager, and a factory worker was formed to develop the questionnaire used in the current study. The questionnaire was pilot-tested among 10 factory workers to assess its clarity and readability. These 10 workers did not participate in the actual survey. Based on the participants’ comments, the panel revised and finalized the questionnaire.

#### Self-Reported Compliance With Personal Preventive Measures in the Past Month

Participants were asked to report the frequency at which they wore face masks in the workplace and in other public settings (public places or transportation) in the past month (response categories: every time, often, sometimes, never). A composite variable was created representing self-reported consistent wearing of a face mask in public places (referring to participants who reported always using a face mask both in the workplace and in other public settings). The participants were also asked what types of face mask they used and whether they reused their face masks. The participants also reported the frequency at which they sanitized their hands using soaps, liquid soaps, and alcohol-based sanitizers after returning from public spaces or touching public installations and equipment (eg, handrails, escalator control panels, or door knobs; response categories: every time, often, sometimes, never), and whether they avoided social meals and gatherings with people who do not live together or avoided crowded places in the past month.

#### Background Characteristics

Participants were asked to report sociodemographic characteristics such as age, gender, internal migrant status, highest education level, relationship status, monthly personal income, status as frontline workers or management staff, and the type of factory they worked in.

#### Individual-Level Variables

To assess the participants’ knowledge related to transmission routes of COVID-19, a composite indicator variable was constructed by counting the number of correct responses to five knowledge items related to COVID-19 transmission routes (ranging from 0 to 5).

To assess their perceptions related to COVID-19, four scales were constructed for this study: (1) the 4-item Perceived Severity Scale, (2) the 4-item Perceived Effectiveness of Individual Preventive Measures Scale, (3) the 2-item Perceived Effectiveness of Governmental Preventive Measures Scale, and (4) the 2-item Perceived Preparedness Scale (preparedness of the health system and workplace). The response categories for these scales were 1=disagree/ineffective, 2=neutral, and 3=agree/effective. The Cronbach alpha values of these four scales ranged from .70 to .92, and single factors were identified by exploratory factor analysis (EFA) that explained 77.3% to 80.9% of the total variance. In addition, a single item was used to measure the participants’ perceived risk of contracting COVID-19 in the next three months (response categories: 1=low, 2=moderate, 3=high), and another item measured the perceived effectiveness of preventive measures implemented by the factories (response categories: 1=very ineffective, 2=ineffective, 3=neutral, 4=effective, 5=very effective).

Depressive symptoms were measured by a validated Chinese version of the Patient Health Questionnaire-9 (PHQ-9) [25]. The Cronbach alpha of the PHQ-9 was .90; one factor was identified by EFA that explained 54.7% of the total variance.

#### Interpersonal-Level Variables

Three items were used to assess the daily average time (hours) of exposure to COVID-19–specific information through official media sources (television, newspapers, and official web-based media such as news apps or blogs and social media accounts of governmental organizations). The Exposure Through Official Media Channels Scale was formed by summing the individual item scores. The Cronbach alpha of the Exposure Through Official Media Channels Scale was .71; one factor was identified by EFA that explained 63.4% of the total variance. In addition, two single items measured the daily average time of exposure to COVID-19–specific information through unofficial media channels (individual blogs and social media accounts) and direct interpersonal communication. The response categories for the aforementioned items were 1=almost none, 2=less than 1 hour, 3=1-2 hours, 4=3-4 hours, and 5=>4 hours.

#### Social-Structural–Level Variables

Both the designated coordinators responsible for COVID-19 control within the sampled factories and the study participants were asked to report whether their factory had implemented seven preventive measures advocated by the Shenzhen government [2,3]. A composite indicator variable was constructed by counting the number of preventive measures implemented by the factory (ranging from 0 to 7). The items and scales measuring individual-level, interpersonal-level, and social-structural–level variables are shown in Multimedia Appendix 1.

### Sample Size Planning

The target sample size was 3000. Given a statistical power of .80 and an alpha value of .05 and assuming the self-reported level of compliance with a personal preventive measure in the reference group (without a facilitating condition) to be 30%-80%, the sample size could detect a smallest odds ratio (OR) of 1.23 between people with and without the facilitating conditions (PASS 11.0, NCSS LLC). Assuming the response rate was 60%, it was necessary to invite 5000 workers to participate in the survey. The median number of workers who had resumed working in factories by the end of February 2020 was approximately 350. Therefore, the research team selected 14 factories for the study.

### Statistical Analysis

Self-reported consistent face mask wearing in public spaces, sanitizing hands every time after returning from public spaces or touching public installations or equipment, avoiding social and meal gatherings with people who do not live together, and avoiding crowded places were the dependent variables. Multilevel logistic regression models (level 1: factories; level 2: individual participants) were fit to analyze the factors associated with the dependent variables. Random intercept models were used to allow the intercept of the regression model to vary across factories, which could account for intracorrelated nested data. Multilevel logistic regression models are commonly used in studies using cluster sampling methods [26]. Univariate two-level logistic models were first used to assess the significance of the association between each of the background characteristics and the dependent variables. Background characteristics with *P*<.05 in the univariate analysis were adjusted in the multivariate two-level logistic regression models. In addition, principal component analysis with varimax rotation was used to perform EFA [27]. SPSS version 23.0 for Windows (IBM Corporation) was used for the data analysis, and *P*<.05 was considered to be statistically significant.

## Results

### Background Characteristics

Of 4158 workers (between 90 and 835 across different factories) who had resumed work in the selected factories on March 1, 2020, 3035 completed the web-based survey (between 56 and 635 participants across different factories); the overall response rate was 73.0%. Over half the 3035 participants were aged ≤30 years (1552, 51.1%), male (1612, 53.1%), internal migrants (2956, 97.4%), married (1812, 59.7%), had not received tertiary education (2004, 66%), had a monthly income lower than ¥5000 (US $714) (1538, 50.8%), were frontline workers (1847, 60.9%), and were manufacturing electronic devices (2353, 77.5%) (Table 1).

**Table 1:** Background characteristics of the participants (N=3035), n (%).

Characteristic		Value
**Age (years)**		
	18-25	653 (21.5)
	26-30	899 (29.6)
	31-40	1195 (39.4)
	>40	288 (9.5)
**Gender**		
	Male	1612 (53.1)
	Female	1423 (46.9)
**Internal migrant**		
	Yes	2956 (97.4)
	No	79 (2.6)
**Relationship status**		
	Single	878 (28.9)
	Have a stable boyfriend or girlfriend	345 (11.4)
	Married	1812 (59.7)
**Highest education level attained**		
	Junior high school or below	1163 (38.3)
	Senior high school or equivalent	841 (27.7)
	College or university	895 (29.5)
	Postgraduate	136 (4.5)
**Monthly personal income (¥)^a^**		
	<3000	175 (5.9)
	3000-4999	1363 (44.9)
	5000-6999	763 (25.1)
	7000-9999	327 (10.8)
	≥10,000	403 (13.3)
**Type of work**		
	Frontline worker	1847 (60.9)
	Manager	1188 (39.1)
**Type of factory worked in**		
	Electronic device manufacturing	2353 (77.5)
	Watchmaking	307 (10.1)
	Beverage manufacturing	191 (6.3)
	Biotechnology product manufacturing	184 (6.1)

^a^1 ¥=US $0.14 on March 1, 2020.

### Self-Reported Compliance With Personal Preventive Measures in the Past Month

In the past month, 2938/3035 participants (96.8%) reported always wearing a face mask in the workplace, and 2996/3035 participants (98.7%) reported always wearing a face mask in other public settings. More than 95% of participants (2904/3035, 95.7%) reported consistently wearing a face mask in any public place. Nonsurgical grade respirators were most commonly used by participants (2073/3035, 68.3%), and 601/3035 (19.8%) reused face masks. Self-reported sanitizing of hands (2152/3035, 70.9%), avoiding social and meal gatherings (2225/3035, 73.3%) and avoiding crowded places (1997/3035, 65.8%) were less common (Table 2).

Table 3, Table 4, and Table 5 show the responses to the survey items measuring the individual-, interpersonal-, and social-structural–level variables, respectively.

**Table 2:** Self-reported compliance with personal preventive measures related to COVID-19 (N=3035), n (%).

Measure and responses		Value
**Frequency of face mask wearing in the workplace**		
	Every time	2996 (98.7)
	Often	33 (1.1)
	Sometimes	3 (0.1)
	Never	3 (0.1)
**Frequency of face mask wearing in public places other than the workplace or on public transportation**		
	Every time	2938 (96.8)
	Often	91 (3.0)
	Sometimes	3 (0.1)
	Never	3 (0.1)
**Consistent face mask wearing in any public space**		
	No	131 (4.3)
	Yes	2904 (95.7)
**Type of face mask worn**		
	Surgical mask	1360 (44.8)
	Nonsurgical grade respirator	2073 (68.3)
	N-95 mask	801 (26.4)
	Cloth mask	161 (5.3)
**Reuse of face masks**		
	No	2434 (80.2)
	Yes	601 (19.8)
**Frequency of hand sanitation (using soap, liquid soap, or alcohol-based sanitizer) after returning from public spaces or touching public installations**		
	Every time	2152 (70.9)
	Often	419 (16.8)
	Sometimes	243 (8.0)
	Never	131 (4.3)
**avoiding social and meal gatherings with other people** **who do not live together**		
	No	810 (26.7)
	Yes	2225 (73.3)
**Avoiding crowded places**		
	No	1056 (34.8)
	Yes	1997 (65.8)

**Table 3:** Responses to survey items measuring individual-level variables (N=3035).

Variable			Value	
**Knowledge about transmission route of COVID-19**				
	**Knowledge about transmission route of COVID-19 (answered Yes), n (%)**			
		Contact with droplets		2871 (94.6)
		Touching contaminated objects		2707 (89.2)
		Direct contact with wildlife Contact with feces		2625 (86.5)
				2364 (77.9)
		Contact with asymptomatic patients		2319 (76.4)
	**Correct responses to COVID-19 transmission route questions**			
		Number of correct responses to COVID-19 transmission route questions, mean (SD)		4.2 (1.3)
		0 correct responses, n (%)		131 (4.3)
		1 correct response, n (%)		49 (1.6)
		2 correct responses, n (%)		94 (3.1)
		3 correct responses, n (%)		264 (8.7)
		4 correct responses, n (%)		634 (20.9)
		5 correct responses, n (%)		1863 (61.4)
**Perceptions related to COVID-19**				
	Perceived risk of contracting COVID-19 (answered High), n (%)		36 (1.2)	
	Perceived risk of contracting COVID-19, mean (SD)		1.3 (0.5)	
	**Perceived consequences of COVID-19 (answered Agree), n (%)**			
		Permanent bodily damage to infected people		1226 (40.4)
		High mortality rate of infected people		1687 (55.6)
		Lack of effective treatment		1687 (55.6)
		Lack of effective vaccines for prevention		1772 (58.4)
	Perceived Severity Scale^a^ score, mean (SD)		9.1 (2.1)	
	**Perceived effectiveness of individual-level preventive measures (answered Effective), n (%)**			
		Wearing face masks		2407 (79.3)
		Sanitizing hands frequently		2464 (81.2)
		Household disinfection		2331 (76.8)
		Avoiding gatherings		2722 (89.7)
	Perceived Effectiveness of Individual Preventive Measures Scale^b^ score, mean (SD)		11.1 (1.8)	
	Perceived effectiveness of preventive measures taken by the factory (answered Effective or Very effective), n (%)		2525 (83.2)	
	Perceived effectiveness of preventive measures taken by the factory, mean score (SD)		4.2 (1.0)	
	**Perceived effectiveness of governmental preventive measures (answered Effective), n (%)**			
		Closure of public spaces (eg, restaurants, theaters)		2610 (86.0)
		Restricting people coming in and out of Shenzhen		2583 (85.1)
Perceived Effectiveness of Governmental Preventive Measures Scale^c^ score, mean (SD)			5.6 (0.9)	
	**Perceived organizational preparedness for COVID-19 outbreak after work resumption (answered Agree), n (%)**			
		The factory in which you are working is well prepared for a COVID-19 outbreak after work resumption		2586 (85.2)
		The medical system in Shenzhen is well prepared for a COVID-19 outbreak after work resumption		2297 (75.7)
Perceived Preparedness Scale^d^ score, mean (SD)			5.6 (0.8)	
**Mental health status**				
	PHQ-9^e^ score, mean (SD)		2.1 (4.0)	
	Probable depression (PHQ-9 score ≥10), n (%)		170 (5.6)	

^a^Perceived Severity Scale: 4 items, Cronbach α=0.70; 1 factor was identified by exploratory factor analysis explaining 77.3% of the total variance.

^b^Perceived Effectiveness of Individual-Level Preventive Measures Scale: 4 items, Cronbach α=.92; 1 factor was identified by exploratory factor analysis explaining 80.9% of the total variance.

^c^Perceived Effectiveness of Structural-Level Preventive Measures Scale: 2 items, Cronbach α=.85.

^d^Perceived Organizational Preparedness Scale: 2 items, Cronbach α=.76.

^e^PHQ-9: Patient Health Questionnaire-9, 9 items, Cronbach α=.90; 1 factor was identified by exploratory factor analysis explaining 54.7% of the total variance.

**Table 4:** Responses to items measuring interpersonal-level variables (N=3035).

Variable			Value	
**Daily average time of exposure to COVID-19–related information through different official media channels, n (%)**				
	**Television**			
		Almost no exposure	613 (20.2)	
		<1 hour	1408 (46.4)	
		1-2 hours	607 (20.0)	
		3-4 hours	146 (4.8)	
		>4 hours	258 (8.5)	
	**Newspapers**			
		Almost no exposure	1627 (53.6)	
		<1 hour	907 (29.9)	
		1-2 hours	294 (9.7)	
		3-4 hours	79 (2.6)	
		>4 hours	127 (4.2)	
	**Official web-based media (news apps, blogs of governmental organizations)**			
		Almost no exposure	134 (4.4)	
		<1 hour	1263 (41.6)	
		1-2 hours	911 (30.0)	
		3-4 hours	258 (8.5)	
		>4 hours	469 (15.5)	
Exposure Through Official Media Channels Scale^a^ score, mean (SD)			7.0 (2.6)	
**Daily average time of exposure to COVID-19–related information through unofficial media channels (eg, personal blogs)**				
	Hours of exposure, mean (SD)			2.4 (1.1)
	Almost no exposure, n (%)			543 (17.9)
	<1 hour, n (%)			1436 (47.3)
	1-2 hours, n (%)			571 (18.8)
	3-4 hours, n (%)			185 (6.1)
	>4 hours, n (%)			300 (9.9)
**Daily average time of exposure to COVID-19–related information through face-to-face communication**				
	Hours of exposure, mean (SD)			1.9 (1.0)
	Almost no exposure, n (%)			1269 (41.8)
	Less than 1 hour, n (%)			1260 (41.5)
	1-2 hours, n (%)			310 (10.2)
	3-4 hours, n (%)			76 (2.5)
	>4 hours, n (%)			121 (4.0)

^a^Exposure Through Official Media Channels Scale, 3 items, Cronbach α=.71; 1 factor was identified by exploratory factor analysis explaining 63.4% of the total variance.

**Table 5:** Responses to items measuring social-structural–level variables (n=3035), n (%).

Preventive measures implemented by the factory	Factory workers (answered Yes)	People responsible for COVID-19 control (answered Yes)
Mandatory 14-day quarantine for employees returning from high-risk areas	2901 (95.6)	14 (100.0)
Prohibiting nonemployees from entering the workplace	2664 (87.8)	14 (100.0)
Taking body temperature and requiring hand sanitation for all employees entering the workplace	2980 (98.2)	14 (100.0)
Providing face masks to all employees	2999 (98.8)	14 (100.0)
Requiring employees to wear face masks in the workplace	3023 (99.6)	14 (100.0)
Frequent workplace disinfection	2986 (98.4)	14 (100.0)
Setting up partitions in factory canteens	2838 (93.5)	14 (100.0)

### Factors Associated With Self-Reported Compliance With Personal Preventive Measures in the Past Month

In the univariate multilevel logistic regression analysis, age, gender, education level, monthly personal income, status as frontline workers or management staff, and type of factory the participants were working in were significantly associated with self-reported compliance with one or more personal preventive measures (Table 6).

**Table 6:** Associations between background characteristics and self-reported compliance with different personal preventive measures.

Characteristic		Wearing a face mask consistently in any public space			Sanitizing hands every time after returning from public spaces or touching installations			Avoiding social and meal gatherings with people who do not live together			Avoiding crowded places	
		OR^a^ (95% CI)	*P* value	OR (95% CI)		*P* value	OR (95% CI)		*P* value	OR (95% CI)		*P* value
**Age (years)**												
	18-25	Reference	N/A^b^	Reference		N/A	Reference		N/A	Reference		N/A
	26-30	1.31 (0.77-2.20)	.32	1.17 (0.94-1.46)		.17	1.10 (0.87-1.39)		.42	1.16 (0.93-1.44)		.19
	31-40	1.29 (0.78-2.12)	.33	1.22 (0.98-1.52)		.07	1.23 (0.98-1.54)		.08	1.27 (1.02-1.57)		.03
	>40	0.51 (0.29-0.91)	.02	1.34 (0.95-1.88)		.09	1.04 (0.75-1.44)		.81	1.18 (0.86-1.60)		.30
**Gender**												
	Male	Reference	N/A	Reference		N/A	Reference		N/A	Reference		N/A
	Female	0.83 (0.58-1.19)	.31	1.20 (1.01-1.41)		.04	0.71 (0.60-0.84)		<.001	0.73 (0.62-0.85)		<.001
**Internal migrants**												
	Yes	Reference	N/A	Reference		N/A	Reference		N/A	Reference		N/A
	No	0.86 (0.33-2.26)	.76	1.55 (0.87-2.79)		.14	1.29 (0.72-2.31)		.39	1.40 (0.81-2.43)		.23
**Relationship status**												
	Single	Reference	N/A	Reference		N/A	Reference		N/A	Reference		N/A
	Having a stable boyfriend or girlfriend	1.28 (0.66-2.48)	.46	1.04 (0.80-1.36)		.77	1.11 (0.83-1.48)		.50	1.13 (0.85-1.48)		.40
	Married	1.10 (0.73-1.64)	.66	1.30 (1.08-1.57)		.005	1.16 (0.95-1.40)		.14	1.11 (0.93-1.33)		.26
**Highest education level attained**												
	Junior high or below	Reference	N/A	Reference		N/A	Reference		N/A	Reference		N/A
	Senior high or equivalent	2.47 (1.53-4.01)	<.001	1.12 (0.91-1.38)		.29	1.64 (1.35-2.00)		<.001	1.77 (1.47-2.13)		<.001
	College or university	2.80 (1.64-4.77)	<.001	0.94 (0.75-1.18)		.59	3.38 (2.66-4.29)		<.001	4.63 (3.70-5.80)		<.001
	Postgraduate	3.69 (1.07-12.71)	.04	1.19 (0.78-1.82)		.42	28.58 (8.94-91.36)		<.001	11.50 (6.04-21.87)		<.001
**Monthly personal income (¥)^c^**												
	<3000	Reference	N/A	Reference		N/A	Reference		N/A	Reference		N/A
	3000-4999	0.84 (0.41-1.73)	.64	1.16 (0.83-1.64)		.39	1.27 (0.92-1.75)		.15	1.40 (1.02-1.93)		.04
	5000-6999	1.82 (0.81-4.07)	.15	1.25 (0.87-1.79)		.24	1.71 (1.21-2.42)		.002	2.16 (1.54-3.03)		<.001
	7000-9999	3.58 (1.21-10.59)	.02	0.89 (0.60-1.34)		.59	3.84 (2.46-5.99)		<.001	4.62 (3.05-7.02)		<.001
	≥10,000	2.04 (0.81-5.10)	.13	1.32 (0.89-1.97)		.17	7.36 (4.54-11.92)		<.001	8.26 (5.32-12.82)		<.001
**Type of work**												
	Frontline worker	Reference	N/A	Reference		N/A	Reference		N/A	Reference		N/A
	Manager	1.69 (1.13-2.52)	.01	1.04 (0.88-1.23)		.66	2.37 (1.96-2.86)		<.001	2.65 (2.22-3.15)		<.001
**Type of factory**												
	Electronic device manufacturing	Reference	N/A	Reference		N/A	Reference		N/A	Reference		N/A
	Watchmaking	2.06 (0.90-4.76)	.09	1.94 (1.44-2.61)		<.001	0.70 (0.55-0.91)		.006	0.63 (0.50-0.80)		<.001
	Beverage manufacturing	0.45 (0.26-0.78)	.005	0.94 (0.69-1.29)		.70	0.94 (0.67-1.30)		.70	0.76 (0.56-1.02)		.07
	Biotechnology product manufacturing	0.40 (0.24-0.69)	.001	2.15 (1.45-3.17)		<.001	1.85 (1.24-2.76)		<.001	2.30 (1.57-3.37)		<.001

^a^OR: odds ratio; crude ORs obtained from two-level logistic regression models (level 1: factories, level 2: individual participants).

^b^N/A: not applicable.

^c^1 ¥=US $0.15.

After adjusting for these significant background characteristics, knowledge about transmission routes of COVID-19 (adjusted odds ratios [AORs] from 1.16, CI 1.10-1.24, to 1.29, CI 1.21-1.37), perceived risk of contracting COVID-19 (AORs from 0.58, CI 0.50-0.68, to 0.85, CI 0.72-0.99), perceived effectiveness of individual (AORs from 1.05, CI 1.00-1.10, to 1.09, CI 1.04-1.13) and governmental (AORs from 1.14, CI 1.04-1.25, to 1.21, CI 1.02-1.42) preventive measures, and the number of preventive measures implemented by the factory (AORs from 1.30, CI 1.08-1.57, to 1.63, CI 1.45-1.84) were associated with self-reported compliance with all four personal preventive measures. Perceived preparedness for a potential outbreak after work resumption was associated with self-reported compliance with all personal preventive measures (AORs from 1.10, CI 1.00-1.21, to 1.50, CI 1.36-1.64), with the exception of consistent wearing of a face mask. Depressive symptoms were associated with consistent wearing of a facemask and self-reported sanitizing of hands (AORs of 0.96, CI 0.92-0.99, and 0.93, CI 0.91-0.94). Perceived severity of COVID-19 was associated with higher self-reported compliance with two physical distancing measures (AORs of 1.05, CI 1.01-1.09, and 1.07, CI 1.03-1.11) but not with consistent face mask wearing or sanitizing hands. In addition, the perceived effectiveness of preventive measures implemented by the factory (AOR 1.30, CI 1.20-1.41), and exposure to COVID-19–specific information through official media channels (AOR 1.08, CI 1.04-1.11) and face-to-face communication (AOR 0.90, CI 0.83-0.98) were associated with self-reported sanitizing of hands but not with other personal preventive measures (Table 7).

**Table 7:** Factors associated with self-reported compliance with different personal preventive measures.

Factor				Consistent face mask wearing in any public spaces				Sanitizing hands every time after returning from public spaces or touching installations			Avoiding social/meal gathering with people who do not live together				Avoiding crowded places			
			AOR^a^ (95% CI)		*P* value		AOR (95% CI)		*P* value		AOR (95% CI)		*P* value		AOR (95% CI)		*P* value	
**Individual-level variables**																		
	**Knowledge and perception**																	
		Knowledge about transmission routes of COVID-19	1.21 (1.08-1.36)		.001		1.16 (1.10-1.24)		<.001		1.18 (1.11-1.26)		<.001		1.29 (1.21-1.37)		<.001	
		Perceived risk of contracting COVID-19	0.71 (0.50-0.99)		.045		0.58 (0.50-0.68)		<.001		0.85 (0.72-0.99)		.047		0.81 (0.69-0.95)		.01	
		Perceived severity of COVID-19	1.03 (0.95-1.12)		.46		1.03 (0.99-1.07)		.09		1.05 (1.01-1.09)		.04		1.07 (1.03-1.11)		.001	
		Perceived effectiveness of individual preventive measures	1.08 (1.00-1.18)		.048		1.09 (1.04-1.13)		<.001		1.06 (1.01-1.11)		.01		1.05 (1.00-1.10)		.03	
		Perceived effectiveness of preventive measures taken by the factories	1.00 (0.83-1.20)		.97		1.30 (1.20-1.41)		<.001		1.01 (0.92-1.10)		.87		1.00 (0.92-1.09)		.98	
		Perceived effectiveness of governmental preventive measures	1.21 (1.02-1.42)		.03		1.14 (1.04-1.24)		.003		1.15 (1.05-1.26)		.004		1.14 (1.04-1.25)		.004	
		Perceived organizational preparedness	0.92 (0.72-1.16)		.47		1.50 (1.36-1.64)		<.001		1.12 (1.02-1.24)		.03		1.10 (1.00-1.21)		.049	
	**Mental health status**																	
		PHQ-9	0.96 (0.92-0.99)		.02		0.93 (0.91-0.94)		<.001		1.01 (0.99-1.03)		.43		1.00 (0.98-1.02)		.66	
**Interpersonal-level variables**																		
	Exposure through official media channels			1.02 (0.96-1.10)		.51		1.08 (1.04-1.11)		<.001		1.00 (0.97-1.03)		.89		1.00 (0.97-1.03)		.80
	Exposure through unofficial media channels			1.03 (0.88-1.21)		.70		1.07 (0.99-1.15)		.13		0.99 (0.92-1.07)		.77		0.99 (0.92-1.06)		.72
	Exposure through face-to-face communication			1.12 (0.92-1.37)		.25		0.90 (0.83-0.98)		.003		1.00 (0.92-1.09)		.99		1.02 (0.94-1.10)		.70
**Social-structural–level variable**																		
	Number of preventive measures implemented by the factory			1.30 (1.08-1.57)		.006		1.63 (1.45-1.84)		<.001		1.34 (1.19-1.51)		<.001		1.47 (1.30-1.66)		<.001

^a^AOR: adjusted odds ratio; background characteristics with *P*<.05 in the univariate analysis were adjusted in the multivariate two-level logistic regression models (level 1: factories, level 2: individual participants).

## Discussion

### Principal Findings

A recent study suggested that physical distancing and population behavioral changes that have a less disruptive economic impact than total lockdown can be effective in controlling COVID-19 [28]. Our study showed that both factories and workers in Shenzhen were well prepared for work resumption. The prevalence of consistent face mask wearing surged from 60% in the early phase of the COVID-19 outbreak (February 2020) [21] to over 95% in our study. Consistent face mask wearing is especially important in workplaces such as factories, where physical distancing cannot be guaranteed. It is also encouraging to see that all the sampled factories proactively implemented all preventive measures advocated by the government [2,3]. These efforts by factories and workers may contribute to effective COVID-19 control after work resumption in China [1].

However, this study highlighted issues related to personal preventive measures that should be addressed by future interventions. First, many workers used non–surgical-grade respirators or even cloth masks, and approximately 20% (601/3035, 19.8%) had reused face masks in the past month. This is understandable, as surgical-grade masks, which provide the highest level of protection against COVID-19, were in limited supply in the early phase of the COVID-19 outbreak in China. To address the supply issue, China has rapidly increased its face mask production capacity. Second, there is a need to improve adherence to hand hygiene and physical distancing measures. Despite WHO recommendations on hand hygiene [7], only 2152 of the 3035 study participants (70.9%) always sanitized their hands. There are some possible explanations for the relatively low adoption of this preventive measure. The importance of hand hygiene may have been less emphasized than consistent face mask wearing in China during the outbreak. Moreover, there may be a lack of appropriate places for workers to sanitize their hands. Only 70% of the factory workers avoided social meals and gatherings or crowded places in the past month. Most Chinese cities enforced community lockdown in the early phase of the outbreak. Some voluntary physical distancing measures will be relaxed when this lockdown is lifted. Without strengthening of preventive measures, local infection is likely to occur.

Our findings provide empirical insights to inform intervention development and suggest the need to tailor interventions to specific groups. Male factory workers were less likely to sanitize their hands frequently but were more likely to comply with physical distancing measures. Promotion efforts should account for gender differences. More attention should be given to workers with lower education levels, as they showed lower compliance with consistent face mask wearing and physical distancing measures compared to workers with higher levels of education. Health communication messages should be straightforward and written at appropriate literacy levels. Management staff performed better in complying with personal preventive measures than frontline workers. These results may be due to the fact that unlike management staff, who primarily work in offices, frontline workers may face barriers to compliance related to their duties and working environment. It is important for factories to identify and address these barriers and enable workers to take necessary precautions. Moreover, the level of self-reported compliance with personal preventive measures varied across different types of factories. Different compositions of workers may explain some of these differences. For example, compared to electronic device manufacturers, workers in watchmaking factories reported higher compliance with hand hygiene but poorer compliance with physical distancing. This difference may be due to the higher proportion of female workers in watchmaking factories (over 70% in this study) compared to that in electronic device manufacturing facilities (approximately 50%). Therefore, interventions should be tailored to different types of factories. Interventions targeting watchmaking factories should focus on physical distancing, while those targeting beverage producers and biotechnology product manufacturers should emphasize consistent face mask wearing.

At the social-structural level, the preventive measures implemented by the sampled factories played important roles in COVID-19 prevention, as knowledge of more preventive measures implemented by the factories was positively associated with compliance with all four personal preventive measures. Some of these measures directly increase access to face masks and facilitate physical distancing (eg, establishing partitions in factory canteens). Moreover, factories can cultivate widely shared organizational norms to facilitate behavioral changes among the workers when implementing these preventive measures [29]. Factories should disseminate these measures to all workers and monitor the implementation of these preventive measures regularly during the pandemic.

Consistent with findings of previous studies, knowledge and perceptions related to COVID-19 had a strong influence on compliance with personal preventive measures [15-18]. Most workers were knowledgeable about transmission routes of COVID-19. New findings such as the risk of transmission among asymptomatic patients or possible fecal-oral transmission should be disseminated to the workers. Compared to results of other studies during the early phase of the outbreak, fewer participants perceived a high risk of contracting COVID-19, probably due to the initial control of the pandemic in China [14]. One possible explanation for the observed negative association between risk perception and compliance may be that people who were not able to comply with these behaviors would perceive higher risk.

Increasing the knowledge and perceived severity of COVID-19 and disseminating the efficacy of individual and governmental preventive measures may be useful strategies in future programs. To enhance compliance with these preventive measures, governments and factories should make their preparedness plans transparent to factory workers. The significant association between perceived effectiveness of preventive measures implemented by the factories and hand sanitation appears to support our speculation that facilities for sanitizing hands in the workplace are an important determinant. Strategically placing hand sanitizer in high-traffic locations throughout the workplace should be considered. Noncompliance with personal preventive measures may be used as a negative coping response to depressive symptoms [30]. Providing psychological support to workers during work resumption is also useful to enhance their compliance with personal preventive measures.

We also found that exposure to different types of media had differing effects on compliance with personal preventive measures, as our results showed that media exposure only influenced hand hygiene. Moreover, exposure through official media channels had a positive impact on hand hygiene, while exposure through unofficial media channels and face-to-face communication had no impact or even a negative impact on the same behavior. Previous studies suggested that the more people read newspapers and watched television reports about MERS, the more knowledge they acquired about the disease and its prevention strategies [22,23]. Compared to official media channels, which mainly report information verified by expert sources, unofficial web-based media channels and face-to-face communication can disseminate not only knowledge but also false or unverified information during a crisis. The null effects of exposure through unofficial web-based media channels may have resulted from conflicting content. The consequences of misinformation can be long-lasting and should not be underestimated in health crisis management [31]. We speculate that hand sanitation was impacted not only by individual perceptions but also by peers’ practices. Because hand sanitation was not highly prevalent, factory workers may have discouraged others from engaging in this behavior during face-to-face communication. Future studies should verify our speculation with a robust examination.

Our study was one of the first studies targeting factory workers at the beginning of work resumption during the COVID-19 pandemic. We used the socio-ecological model as a theoretical framework and examined potential associated factors at multiple levels. This study provides evidence to inform multilevel programs to strengthen compliance with personal preventive measures among factory workers. Currently, many countries are in the early stage of work resumption and are attempting to achieve a balance of economic reactivation and COVID-19 pandemic control; our findings have some reference value for these countries.

### Limitations

This study has some limitations. First, policies and guidelines related to COVID-19 control are being updated rapidly in response to the quickly changing pandemic. These changes in national policies and guidelines have strong influences on self-reported compliance with personal preventive measures. For example, the National Health Commission of China updated the requirement to wear a face mask in the workplace on March 18, 2020, stating that face mask wearing is required in the workplace only when people are in close contact with others (<1 meter). Therefore, our findings are most applicable to the early phase of the COVID-19 outbreak, when strict measures were enforced, and have limited implication for the current situation in China. However, the risk of a second wave of COVID-19 still exists in China. In the case of another wave, some strict control measures are likely to be implemented again. Our findings could inform effective interventions facilitating the implementation of these strict control measures. Second, we only included factory workers in one Chinese city. Generalization should be made cautiously to individuals working in other types of enterprises or to other geographic locations in China. Third, because this study was anonymous and participants’ personal contacts and identifying information were not collected, we were not able to collect information from workers who refused to participate in the study. Factory workers who refused to complete the survey may have different characteristics from the participants. Selection bias existed. Our response rate was relatively high (73.0%) compared to other web-based surveys on similar topics [19,20]. Fourth, the data were self-reported, and verification was not feasible. Recall bias may have occurred. Participants may have also overreported their compliance with personal preventive measures due to social desirability. Fifth, most items and scales used in this study were self-constructed based on those used in previous studies on SARS and H1N1 in China [15-18]. The internal reliability of these scales was acceptable; however, they may require external validation. Sixth, we arbitrarily chose the cutoffs for different age groups. Moreover, some behavioral factors that may influence personal preventive behaviors during the COVID-19 pandemic were not considered in this study, such as previous experiences with pandemics, concerns related to personal protective equipment supply, resource constraints, and comfort of adopting these preventive measures [32]. National guidelines emphasize that maintaining good ventilation in the workplace is an essential strategy for COVID-19 control [33]. Failure to consider ventilation in the workplace was another limitation of this study. Furthermore, causality could not be established, as this was a cross-sectional study.

### Conclusions

Factory workers in China self-reported a very high level of compliance with consistent face mask wearing at the beginning of work resumption. However, compliance with hand hygiene and physical distancing measures should be strengthened. Strategically placing hand sanitizer in the workplace should be considered. Future studies should address multilevel factors associated with these preventive measures. Our findings have some reference value for other countries that are in the early stage of work resumption.

## Appendix

Multimedia Appendix 1 Survey items measuring individual-, interpersonal-, and social-structural–level variables in both English and Chinese.

## Abbreviations

AOR: adjusted odds ratio
EFA: exploratory factor analysis
MERS: Middle East respiratory syndrome
OR: odds ratio
PHQ-9: Patient Health Questionnaire-9
SARS: severe acute respiratory syndrome
WHO: World Health Organization
