# Supplementary material for: Self-Reported Compliance With Personal Preventive Measures Among Chinese Factory Workers at the Beginning of Work Resumption Following the COVID-19 Outbreak: Cross-Sectional Survey Study
Source: J Med Internet Res. 2020 Sep 29;22(9):e22457. doi: 10.2196/22457 (PMC7527164; doi:10.2196/22457)
Supplement: Multimedia Appendix 1 [file jmir_v22i9e22457_app1.docx]

Multimedia Appendix 1: Items measuring individual-, interpersonal-, and socio-structural-level variables in both English and Chinese

**Individual-level variables**

1. Knowledge about transmission route of COVID-19

| 据你所知，「新型冠状病毒」……  To your knowledge, whether COVID-19 can be transmitted through …… | 可以  Yes | 不可以  No | 不确定  Uncertain |
| --- | --- | --- | --- |
| A可以经病人飞沫传播 (如：打喷嚏、咳嗽)  Droplets | 1 | 2 | 3 |
| B可以经接触受病毒污染的物件传播  Touching contaminated objects | 1 | 2 | 3 |
| C可以经接触野生动物传播  Direct contact with wildlife | 1 | 2 | 3 |
| D可以经粪便传播  Fecal-borne | 1 | 2 | 3 |
| E可以经没有症状的「新型冠状病毒」感染者传播  Asymptomatic patients | 1 | 2 | 3 |

2. Perceived risk of contracting COVID-19

|  | 低  Low | 中等  Moderate | 高  High |
| --- | --- | --- | --- |
| 在未来三个月内，你自己感染新冠肺炎的机会有多高？  Perceived one’s risk of contracting COVID-19 in the next three months | 1 | 2 | 3 |

3. Perceived severity of COVID-19

| 你是否同意以下有关新冠肺炎的说法？  Do you agree with the following statement related to COVID-19 | 同意  Agree | 中立  Neutral | 不同意  Disagree |
| --- | --- | --- | --- |
| A会很容易导致永久性的身体严重损害  COVID-19 would result in permanent bodily damage among infected people | 1 | 2 | 3 |
| B新冠肺炎的死亡率很高  COVID-19 has high mortality rate of infected people | 1 | 2 | 3 |
| C现在没有已证实的特效药物能有效治疗新冠肺炎  There is a lack of effective treatment for COVID-19 | 1 | 2 | 3 |
| D现在没有疫苗可以有效预防新型冠状病毒  There is a lack of effective vaccines for prevention | 1 | 2 | 3 |

4. Perceived effectiveness of individual-level preventive measures

| 你认为以下的防疫手段有多有效？  Do you think the following measures are effective? | 无效  Ineffective | 中立  Neutral | 有效  Effective |
| --- | --- | --- | --- |
| A佩戴口罩  Wearing facemasks | 1 | 2 | 3 |
| B经常消毒双手  Sanitizing hands frequently | 1 | 2 | 3 |
| C消毒家居  Household disinfection | 1 | 2 | 3 |
| D避免聚集  Avoiding gathering | 1 | 2 | 3 |

5. Perceived effectiveness of preventive measures taken by the factories

|  | 完全无效 | 不太有效 | 中立 | 比较  有效 | 非常有效 |
| --- | --- | --- | --- | --- | --- |
| 总体而言，您觉得您所在企业采取的防疫措施能够有效预防新冠肺炎？  In general, do you think the preventive measures taken by the factory are effective in preventing COVID-19? | 1 | 2 | 3 | 4 | 5 |

6. Perceived effectiveness of governmental preventive measures

| 你认为以下的防疫手段有多有效？  Do you think the following measures are effective? | 无效  Ineffective | 中立  Neutral | 有效  Effective |
| --- | --- | --- | --- |
| A关停公共场所比如餐厅、电影院等  Closure of public spaces (e.g., restaurants, theatres, etc.) | 1 | 2 | 3 |
| B采取对人员出入深圳市的管制措施  Restricting people coming in/out of Shenzhen | 1 | 2 | 3 |

7. Perceived preparedness of health system and workplace

| 你是否同意以下的看法  Do you agree with the following statements? | 同意  Agree | 中立  Neutral | 不同意  Disagree |
| --- | --- | --- | --- |
| A您所在的企业对复工后的疫情控制做好了充足准备  The factory in which you are working is well-prepared for COVID-19 outbreak after work resumption | 1 | 2 | 3 |
| B深圳的医疗系统对复工后的疫情控制做好了充足准备  Medical system in Shenzhen is well-prepared for COVID-19 outbreak after work resumption | 1 | 2 | 3 |

**Interpersonal-level variables**

| 你平均每天通过下列渠道接触新冠肺炎相关信息的总时长为？  Daily average time (hours) of exposure to COVID-19 specific information through the following channels | 几乎  没有  Almost none | 1小时以内  Less than 1 hour | 1-2  小时  1-2 hours | 3-4  小时  3-4 hours | 4小时以上  >4 hours |
| --- | --- | --- | --- | --- | --- |
| A电视节目  Television | 0 | 1 | 2 | 3 | 4 |
| B报纸  Newspaper | 0 | 1 | 2 | 3 | 4 |
| C网上的官方媒体，包括新闻类手机软件、政府机构的（如卫健委、疾病控制中心、人民日报等）网站、微博及公众号  Online official medias (news apps, blogs of governmental organizations) | 0 | 1 | 2 | 3 | 4 |
| D网上的非官方媒体，例如个人的微博帖子、公众号、朋友圈等  Unofficial media channels (e.g., other people’s blogs) | 0 | 1 | 2 | 3 | 4 |
| E与他人面对面交流  Face-to-face communication | 0 | 1 | 2 | 3 | 4 |

**Socio-structural-level variables**

Preventive measures implemented by the factory in which you are working

| 据您所知，你所在的企业是否采取了以下的措施  Did the factory in which you are working implement the following preventive measures? | 有  Yes | 没有  No | 不知道  Uncertain |
| --- | --- | --- | --- |
| A疫情高发地返回深圳的员工要隔离观察14天  Mandatory 14-day quarantine for employees returning from high-risk area | 1 | 2 | 3 |
| B禁止非员工进入工作场所  Prohibiting non-employee entering workplaces | 1 | 2 | 3 |
| C为所有进入工作场所的员工量体温和消毒双手  Taking body temperature and sanitizing hands for all employees entering the workplace | 1 | 2 | 3 |
| D为所有员工提供口罩  Providing facemasks to all employees | 1 | 2 | 3 |
| E要求员工上班期间必须全程佩戴口罩  Requiring employees wearing facemasks in workplace | 1 | 2 | 3 |
| F为工作场所经常消毒  Frequent workplace disinfection | 1 | 2 | 3 |
| G就餐位置之间设置隔板  Setting up partitions in factory canteens | 1 | 2 | 3 |
